# Understanding compassion fatigue among social workers: a scoping review

**DOI:** 10.3389/fpsyg.2025.1500305

**Published:** 2025-01-27

**Authors:** Tian Wu, Chun-Rong Lu

**Affiliations:** ^1^Department of Rehabilitation Sciences, Nanjing Normal University of Special Education, Nanjing, China; ^2^Department of Social Work, Tunghai University, Taichung, Taiwan

**Keywords:** compassion fatigue, trauma, scoping review, social worker, themes analysis

## Abstract

Exposure to significant sources of trauma and stress among social workers is increasingly identified as an important issue that can impact the quality of professional services, and the social worker’s well-being. There is more and more literature focusing on this phenomenon and related concepts. However, to our knowledge, there has been no published systematic review of the literature on compassion fatigue (CF) among social workers. Therefore, we performed a systematic scoping review by searching five electronic databases for studies published from 2001 to 2021, with the aim of identifying relevant literature. A total of twenty-nine studies were selected in the review following the systematic search strategy. Five themes were identified through the use of a narrative approach to synthesizing the literature by a Five-step framework of scoping review, including the prevalence, factors related, attributes and characteristics, consequences and strategies or interventions to reduce compassion fatigue among social workers. Findings revealed that few studies had examined the consequences and tested the effectiveness of specific interventions. Furthermore, future research involving concept analysis and related theoretical model was required in the field of social work.

## Highlights

- Better understanding of social workers’ experiences of compassion fatigue can improve their wellbeing and service delivery.
- Further research is required to fully understand the consequences and interventions on the quality of services provided.
- Future research exploring compassion fatigue across organizational settings, and identifying strategies to mitigate compassion fatigue among social workers would enhance the current knowledge and related theoretical models.

## Introduction

As a practice based profession, the main task of clinical social workers is to establish professional relationships and empathize with people who have experienced or are currently facing multiple traumas. Consequently, social workers in various roles are highly susceptible to experiencing emotional and psychological distress, such as vicarious traumatization (VT), secondary traumatic stress (STS), compassion fatigue (CF) or job burnout (BO), due to prolonged exposure to various types of traumatic realities (Armes et al., 2020; Hamid and Musa, 2017). Research on social workers in a range of settings reported that they were almost at risk of developing compassion fatigue, such as child welfare caseworkers (Rienks, 2020), hospice social workers (Fox, 2019) and social workers in the community settings (Brown et al., 2019). These literatures suggested that compassion fatigue can have multiple negative effects on social workers and even have the negative implications for organizations and service users (Ledoux, 2015). The negative psychological and physical impacts of CF involved hopelessness; deep physical, emotional, and spiritual exhaustion; disconnection from others; and affected the personal and professional well-being of care providers (Figley, 1995). Meanwhile the social workers in high-risk situations will encounter employee absenteeism, turnover, poor morale, and impaired professional judgment (Denne et al., 2019; Kim et al., 2021). Better understanding of social workers’ experiences of compassion fatigue can improve their wellbeing and optimize the efficiency and quality of social services delivered to those in need (Bride et al., 2016; Decker et al., 2002).

Compared to secondary traumatic stress (Sorenson et al., 2016), the term compassion fatigue, which was considered as a user-friendly alternative for the outcome of work-related stress, had been widely adopted after its publication by Joinson in 1992 (Coetzee and Laschinger, 2018). As two different conditions with similar features, secondary traumatic stress and vicarious traumatization located different areas of symptoms, respectively. The former emphasized outward behavioral symptoms and the later focused on intrinsic cognitive changes (Newell and MacNeil, 2010). Since then, significant advancements had been made in the field of compassion fatigue research, particularly in terms of conceptual and methodological domains. These advancements focused on establishing conceptual clarity, conducting empirical research and developing theoretical and measurement models (Bride et al., 2007; Cross, 2019; Sorenson et al., 2017). The existing measurement tools used to assess CF among social workers included the professional quality of life scale (ProQOL) and compassion fatigue scale (CFS).

However, compared with these studies in health care, there was a limited number of studies reviewing the compassion fatigue related concepts in social work (Letson et al., 2020). While Molnar et al. (2020) conducted a review on vicarious traumatic stress in early childhood professionals through the analysis of 39 articles, as far as we know, there has been a lack of systematic review focusing on compassion fatigue specifically among the social workers. This gap in the literature research made it challenging to identify the prevalence of compassion fatigue among social workers and to develop prevention and intervention strategies (Méndez-Fernández et al., 2022).

As a type of knowledge synthesis for literature reviews that has received little attention, scoping reviews are particularly effective in mapping evidence on broader topics (Arksey and O'Malley, 2005; Harms and Goodwin, 2019). Therefore, we conducted a scoping review by utilizing a systematic approach, analyzing and summarizing the scope of findings in current literatures.

The purpose of this scoping review was to

- Identify the quantity and scope of current literature.
- Provide a summary and synthesis of significant research findings.
- Explore the gaps in the available literature to guide future research.

## Methods

In contrast to systematic reviews, scoping reviews preliminary assess the potential scope and extent of available literature through a broad approach to uncover the current knowledge gaps (Arksey and O'Malley, 2005; Grant and Booth, 2009; Harms and Goodwin, 2019). The selection of a scoping review methodology for this study was appropriate to achieve the goals of mapping the current literature and informing the findings for social work practice (Molnar et al., 2020). We employed the scoping review following the framework proposed by Arksey and O'Malley (2005), and Preferred Reporting Items for Systematic Reviews and Meta-Analyses (PRISMA) framework as a systematic approach (Tricco et al., 2018). The review process consisted of the following five stages.

### Stage 1: identifying the research question

What research in English and Chinese language had been undertaken on compassion fatigue among social workers?

### Stage 2: identification of relevant studies

The two-pronged search strategy was utilized. The first strategy involved a systematic literature search of five electronic databases, namely PubMed, ERIC, Web of Science, EBSCO (Psychology and Behavioral Sciences Collection, APA PsycArticles, APA PsyInfo, and Open Dissertations), CNKI, and Airiti (two electronic databases in Chinese), spanning from January 2001 to December 2021. One set of search terms describing participants (“social work” OR “social workers” *) was combined with a second set of keywords describing CF (“compassion fatigue” OR “compassion satisfaction” OR “secondary traumatic stress” OR “burnout”*) in all possible permutations. In addition, to avoid the risk of omitting relevant studies, a hand-search of the reference lists of located studies was also conducted and identified five additional publications.

### Stage 3: selecting studies

A systematic selection process was utilized to determine the final set of articles that reflected the international trends. For inclusion in the review, articles were (a) empirical article published in a peer-reviewed journal, (b) written in English or Chinese, with (c) primary focus on social workers or related professionals, and (d) studied the compassion fatigue. The reasons why certain articles were not considered for this scoping review were as follows: (a) they focused on other professions and did not include information about social workers; or (b) they focused on the related concept other than compassion fatigue (i.e., Secondary Traumatic Stress, Burnout, Professional Resilience). In addition, as a revised version of the compassion fatigue scale (CFS), the professional quality of life scale (ProQOL) was the most commonly used assessment tool, which consisted of three subscales—compassion satisfaction, burnout, and secondary traumatic stress—where compassion fatigue was defined as a combination of burnout, and secondary traumatic stress. Therefore, if the article involved multiple concepts and used the Pro-QOL scale as the main measurement concept, they were included.

**Figure 1:**
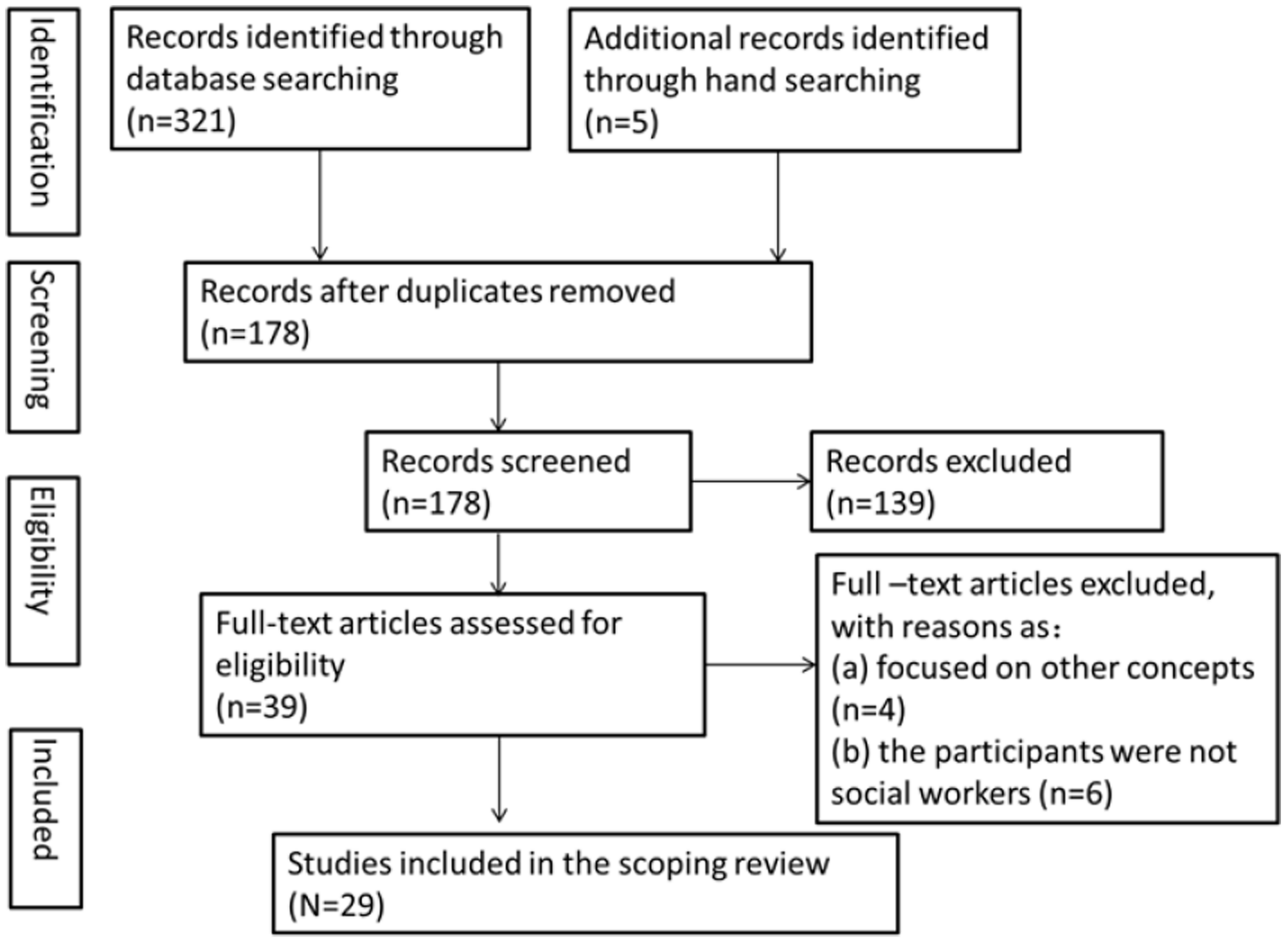
PRISMA flowchart of the study selection process.

Two authors conducted the process independently and then checked agreement. The reliability of the searching was determined by comparing the number of articles identified by the two reviewers. Five articles with different opinions were discussed in more depth between the two reviewers and resolved through reconsidering inclusion to reach a consensus. Figure 1 provided a summary of our search process and results as presented in the PRISMA chart (Tricco et al., 2018).

### Stage 4: data graphics

Two reviewers collaborated to extract descriptive characteristics from the articles, and developed a standardized data extraction form that facilitated the gathering of vital theoretical and methodological elements, including: (a) author (s) and publication year, (b) study location: country and setting, (c) study populations (e.g., child protection social work), (d) purpose of the study, (e) research design and methodology, and (f) instrument. In addition, to illuminate the dominant areas of research, the reviewers identified the outcomes from the included papers. These detailed data extractions can be found in Table 1, which were collated by the reviewers using a customized data extraction sheet.

**Table 1:** Characteristics of the 29 included studies.

Authors, year, and country	Settings	Design	Sample	Research aims	Instrument	Outcomes
Adams et al. (2006) USA	Current members of NASW	Quantitative (Factor analyses)	236 social workers living in New York City	To assessed the construct validity of the CF scale.	CF scale	CF scale measured 2 key dimensions—STS and BO, which to be quite appropriate assessment tools, either separately or combined into the CF-Short Scale.
Adams et al. (2008) USA	Current members of NASW	Quantitative	236 social workers living in NYC	To examine the utility of secondary trauma in predicting psychological distress.	CF Scale	Social workers’ involvement in WTC recovery efforts is related to secondary trauma but not burnout.
Baugerud et al. (2018) Norway	Child Protective Services (CPS’s)	Quantitative	506 CPS workers	To evaluate the presence of STS and BO, as well as levels of CS in CPS workers, and examine risk and protective factors.	Pro-QOL	83.7 per cent of the respondents experienced moderate levels of CS.
Boscarino et al. (2004) USA	Current members of NASW	Quantitative	236 social workers living in New York City (NYC)	To predict ST and BO related to providing services to those affected by the World Trade Center (WTC) attacks.	CF scale	ST was positively associated with WTC involvement and negatively associated with supportive work environment.
Bourassa (2012) USA	APS unit and aging services	Qualitative (Semistructured interview)	9 Adult Protective Services (APS) social workers	To explore the experiences and perspectives of APS social workers in relation to CF.		The APS social workers combined personal characteristics and professional factors to develop boundary-setting mechanisms that protected them from
Brown et al. (2017) USA	Transitional age youth, supportive services and so on	Quantitative	mental health professionals (*n* = 40) and MSW students (*n* = 111)	To explore the relationship between CF and mindfulness in mental health professionals compared to Master of Social Work (MSW) students.	Pro-QOL	Results indicate a medium, negative correlation between CF and mindfulness. There was no statistically significant difference between mental health workers and MSW students.
Conrad and Kellar-Guenther (2006) USA	Child protection settings	Quantitative	363 child protection staff	To determine the prevalence of CF, BO, and CS among child protection workers.	CF scale	Approximately 50% of child protection staff suffered from “high” or “very high” levels of CF. The risk of burnout was considerably lower.
Cuartero and Campos-Vidal (2019) Spain	Social centres	Quantitative	270 social workers	To assess the efficiency of social workers’ self-care practices on CF.	Pro-QOL	Personal and professional self-care practices reduce CF levels as well as increase satisfaction levels.
Denne et al. (2019) USA	Child dependency court	Quantitative	The social workers who working with children (*N* = 173 + *N* = 119)	To explore the relationship between social workers’ CF and years of job experience on hypothetical child custody case judgments.	CF Scale	CF significantly mediated the relationship between increased years of social worker job experience on recommendations that a neglectful mother receive custody
Finzi-Dottan and Kormosh (2016) Israel	In a broad range of contexts	Quantitative	202 social workers	To examine the moderating role of traumatic life events and of self-differentiation and professional self-esteem on CF and on its spillover into marital quality.	Pro-QOL	High CS and professional self-esteem contributed to the participants’ marital quality. Self-differentiation moderated the effect of secondary traumatization, and professional self-esteem moderated the effect of burnout on their marital quality.
Fontin et al. (2020) USA	Community and hospital setting	Quantitative cross-sectional study	93 violence intervention caseworkers	To describe the prevalence of CS and CF among caseworkers.	Pro-QOL	Participants significantly differed in CF by years of experience and workplace setting. Caseworkers who had been working in their profession for 6–10 years experienced higher levels of burnout than those working fewer years. Caseworkers employed in a single program setting experienced significantly lower levels of STS than those who work in both a community and hospital setting.
Fox (2019) Australia	Hospital	Qualitative (narrative, ethnography)	A hospital-based social worker	To investigate the phenomenon across the profession and provide a critique of the needs of practitioners working in the complex environment of hospitals and health care. Parallel		Developed the understandings of the lived experience of compassion fatigue and vicarious trauma on the social work profession.
Geoffrion et al. (2019) Canada	Youth center	Quantitative (Factor analyses)	310 child protection workers	To assessed the construct validity of the ProQOL scale.	Pro-QOL	A bifactor model postulating a factor structure with a general factor in addition to independent factors (CS, bo, and STS) was proposed.
Gregory (2015) Australia	In a broad range of contexts	Quasi-experimental design	11 social workers	To report the effectiveness of a yoga and mindfulness program to decrease CF and to increase cs in currently employed social workers	Pro-QOL	Participation in a brief yoga and mindfulness program may halt the decrease of CS.
Harr et al. (2014) USA	In field placements	Quantitative	Social work students (*n* = 480) and employed professionals (*n* = 186)	To explore the impact of CF and CS on social work students compared with employed professionals.	Pro-QOL	social work students experience lower levels of compassion fatigue than the professionals.
Jacobson et al. (2013) USA	A cluster of Lutheran churches	Quantitative	Ninety-five clergy	To explore the relationship of personal and organizational characteristics, along with symptoms of depression, and CF, burnout, and potential for CS.	Pro-QOL	Clergy and SW were at low risk for burnout and moderate risk for CF and they had a moderate potential for CS, and the years in service and reported depression not predict risk for CF.
Kapoulitsas and Corcoran (2014) Australia	In a community service organization	Qualitative (semi-structured interviews)	6 social workers	To examine what develops personal, professional and organizational resilience; and the ways in which workers can be better protected from CF.		Four major themes were identified: The complexities of social work, Supportive and unsupportive contexts, Promoting personal well-being/self-protection, Resilience as a changing systemic and complex process.
Kiley et al. (2018) USA	A mental health nonprofit organization	Two-arm randomized controlled trial	69 Mental health professionals	To examine the effects of prerecorded guided imagery (GI) on CF and state anxiety.	Pro-QOL	Results revealed statistically significant differences in change scores between the control and experimental groups for state anxiety and sleep quality.
Kim et al. (2021) Korea	Hospitals	Qualitative	12 medical social workers	To explore the impacts of CF on social workers working with oncology patients.		Main themes: Personal wellbeing, Work, Coming to terms with CF, Transforming the impact of CF.
Kreitzer et al. (2020) Canada	Home care, community-based mental health services and so on.	Qualitative (phenomenological methodology)	14 social workers	To explore the institutional factors that social worker’s identified as contributing to their experience of CF.		The factors are presented including: Costeffective services within time constraints and political climates; Erosion of relationship building; Lack of communication between managers and front line workers; Cutbacks in services; Climate of fear; and Outcome measurement requirements.
Letson et al. (2020) USA	A children’s advocacy center (CAC) setting	Quantitative (Cross sectional survey) and Qualitative (open-ended survey questions)	885 child welfare workers	To investigate CS, BO and STS and their associated factors among child abuse professionals working in a CAC setting.	Pro-QOL	Child welfare workers had significantly higher burnout scores and significantly lower CS scores than most others. Professionals providing on-call services had significantly higher burnout.
Pelon (2017) USA	Hospice settings	Quantitative (cross-sectional study)	55 hospice social workers	To explored the prevalence of compassion fatigue among hospice social workers.	Pro-QOL	Hospice social workers exhibited moderate to high levels of CF, they were also found to have moderate to high levels of CS.
Samios (2017) Australia	In a broad range of contexts	Quantitative	69 rural mental health workers	To examine the buffering role of mindfulness on the relationship between burnout and psychological adjustment and test a mechanism through which mindfulness exerts its propitious effects: through CS.	Pro-QOL	CS partially mediated the relationship between mindfulness and depression and fully mediated the relationships between mindfulness and the positive indicators of adjustment.
Thomas (2013) USA	In a broad range of contexts	Quantitative (cross-sectional survey)	171 clinical social workers	To examine the relationship of personal distress and three other aspects of the empathy construct with CF, BO, and CS.	Pro-QOL	Higher personal distress is associated with higher CF and BO and lower CS among clinical social workers, and personal distress is the only component of the empathy construct with significant associations with the dependent variables.
Waegemakers Schiff and Lane (2019) Canada	Homeless shelters	Quantitative	472 frontline workers	To examine the relationship between STS and burnout through a direct measure of PTSD symptom and related factors.		The rates of BO&CF comparable to workers in other social services organizations, but the rates of PTSD symptoms to be at 33% of the total population.
Wagaman et al. (2015) USA	In a broad range of contexts	Quantitative	173 social workers	To explore the relationship between the components of empathy, burnout, STS, and CS.	Pro-QOL	The components of empathy may prevent or reduce burnout and STS while increasing CS.
Yi et al. (2018) USA	Hospital or community-based settings	Qualitative (focus groups)	27 pediatric oncology social workers	To understand the experience of compassion fatigue among 27 pediatric oncology social workers.		Four main themes emerged: Conditions that contribute to CF; The influence of CF; Coping strategies to alleviate CF; and desire for systematic support to prevent CF.
Yi et al. (2016) Korea	Hospital	Qualitative (in-depth qualitative Interviews)	12 medical social workers who providing care for oncology patients	To examine when compassion fatigue is experienced and how it is dealt with by medical social workers.		When CF hits me (when bonding with clients, when facing a client’s death, when facing organizational hurdles, when feeling inadequate) How I deal with compassion fatigue (communicating with others, setting professional boundaries, finding ways to help myself, creating grief rituals, building professional identity).
Yi et al. (2018) Korea	Hospitals	Quantitative (Cross sectional survey)	97 medical social workers	To examine the role of empathy in relation to CS and CF among medical social workers.	Pro-QOL	Empathic concern was positively associated with CS and negatively associated with BO. Personal distress was correlated with CS, STS, and BO.

### Stage 5: summarizing and reporting the results

The results were presented by examining, comparing, synthesizing, and discussing the study characteristics and primary findings of the included articles. A narrative synthesis format was utilized, in which the themes were organized into an inductive conceptual structures to facilitate the discussion of the results.

## Results

Initially, a total of 326 articles were identified through electronic database searching and manual searching. After the removal of duplicates, 178 articles remained and the titles and abstracts of them were screened. 139 articles were excluded in accordance with the inclusion criteria (interrater reliability 96%). Finally, the full texts of the remaining 39 articles were examined to reach a final decision. 10 articles were excluded at full-text review due to the excluded standard (interrater reliability 94%). Figure 1 shows the PRISMA flowchart of the study selection process.

**Figure 2:**
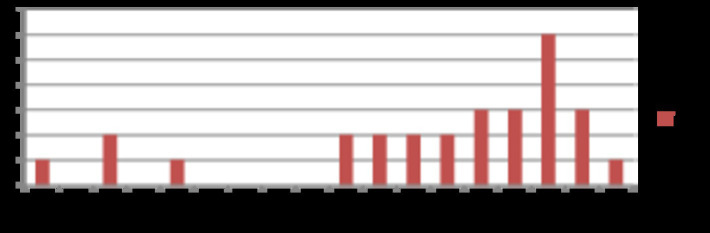
Year of publication of the 29 included studies.

Table 1 presented the general characteristics of these studies. All included articles were in English (*n* = 29). Most of the studies were conducted in the USA (*n* = 16), and followed by Australia (*n* = 4), Canada (*n* = 3), Korea (*n* = 3), Norway (*n* = 1), Spain (*n* = 1) and Israel (*n* = 1). The majority of studies were published between 2013 and 2021 (*n* = 24), reflecting the emerging research status of this field (see Figure 2). Most of the studies employed quantitative methods (*n* = 21) and eight papers used qualitative methods. Two main measurement tools with potential use for CF among social workers across the quantitative studies were identified, including the Compassion Fatigue Scale (*n* = 5) and Professional Quality of Life (ProQOL) scale (*n* = 16). Table 1 listed the characteristics of the methodologies used in all the included studies.

### Theme 1: prevalence of CF among social workers

The existing instruments used to assess compassion fatigue among social workers included the professional quality of life scale (ProQOL) and compassion fatigue scale (CFS) (Keesler and Fukui, 2020). Sixteen of the studies in this review employed the Pro-QOL (III, IV, V) and five used the CFS. Ten studies reported data on the level and prevalence. Overall, most studies (*n* = 7) reported that more than half of the participants scored in the medium range of compassion fatigue. However, Letson et al. (2020) found child welfare workers scored high category for burnout scale (68th percentile) and the top quartile for STS scale (94th percentile). Baugerud et al. (2018) found none of the respondents scoring in the high range of burnout (BO) and secondary traumatic stress (STS), meanwhile some of the participants (32.6%) scored high. (Cuartero and Campos-Vidal, 2019). There was no data available in the Chinese literature.

### Theme 2: factors related to CF in social workers

We coded the factors that correlated with compassion fatigue across the reviewed studies, including the demographic differences (i.e., age, gender, marital status, education), the traits of personality (i.e., empathy, self-care, mindfulness, personal distress) and the work-related factors (i.e., employment status, length of employment, job position, peer Support). Baugerud et al. (2018) reported that the positive challenges at work, a sense of mastery of the work and commitment to their organizations were associated with improved levels of compassion satisfaction. Yi et al. (2018) and Wagaman et al. (2015) both focused on the role of empathy and reported negative correlation with compassion fatigue.

### Theme 3: attributes and characteristics of CF in social workers

To understand the attributes and characteristics of compassion fatigue among social workers, the voices from themselves was very important. Kapoulitsas and Corcoran (2014) identified the emotional responses and difficulties from the interviews involving six social workers who working with distressed clients and Yi et al. (2016) described when compassion fatigue was experienced and how it was dealt with within the oncology social workers. There were two key defining attributes were identified from the relevant literature, including the cumulative process and emotional and behavioral symptoms.

### Theme 4: consequences of CF in social workers

Compared to the studies focusing on the predictors of compassion fatigue, there was limited research on the consequences of compassion fatigue, with only two studies identified in this scoping review. Kim et al. (2021) explored the negative consequences of compassion fatigue on the oncology social workers’ well-being, including the personal wellbeing and transforming the impact of compassion fatigue. Denne et al. (2019) found that compassion fatigue could change the perspective of social workers when dealing with cases of child abuse and Kapoulitsas and Corcoran (2014) provided another insight into the participants’ experiences, like the resilience as the growth factors.

### Theme 5: strategies or interventions to reduce CF among social workers

Among the studies reviewed, two focused on interventions aimed at reducing compassion fatigue in social workers, such as yoga and mindfulness programs and pre-recorded guided imagery (GI). Kiley et al. (2018) provided an evaluation of the effects on compassion fatigue and anxiety among the mental health, while Brown et al. (2017) confirmed that compassion fatigue and mindfulness were inversely correlated. However, there was no formal intervention in these two studies. There were no relevant reports in Chinese language.

## Discussion

This scoping review sought to examine and map a broad spectrum of published literature concerning social workers’ experiences with compassion fatigue. There had been a growing focus on the population of social workers (ex., compared to the nurses), with almost 82.8% of the literature published between 2013 and 2021.

Almost all peer-reviewed papers came from high-income countries, showing that there are regional differences in this research, which was also closely related to the development of the social work profession. Future research needed to obtain research data from more countries and regions around the world, including comparisons evidence between different countries, in order to capture the CF level of social workers around the world in a more comprehensive way. Additionally, five themes of the included literature were identified through the thematic analysis. These themes were also documented in previous reviews that centered on other professionals (Najjar et al., 2009; Ortega-Campos et al., 2019; Zhang et al., 2018). Baqeas et al., 2021 reported the themes emerged from synthesis, including consequences, associated factors and strategies to support palliative care health providers. To some extent, it was similar with this current review. However, our findings revealed that most of the studies investigated the prevalence and related factors, which indicated the gap in the literature that needed more examination.

Firstly, a concept analysis and related attributes or theoretical model needed to be discussed and a more suitable measurement tool for social workers needed to be developed. Based on our review, the findings indicated that there was no existing work provided a concept analysis of compassion fatigue among the social workers. However, there were some researches that focused on the concept analysis of compassion fatigue among the healthcare providers or the family caregivers in the healthcare setting (Cross, 2019; Lynch and Lobo, 2012). A concept definition of compassion fatigue in the context of social work could provide more insight for this profession and facilitate the identification of symptoms and risk factors among social workers (Coetzee and Laschinger, 2018). Most of the included studies used the ProQOL scale, which is widely used among the nurses and medical staff (Ortega-Campos et al., 2019). Future research should aim to enhance theoretical understanding of compassion fatigue and develop a more suitable measurement tool for social workers.

Second, this scoping review revealed the CF-related risk and protective factors in social workers across the reviewed studies. The main factor that affected social workers in terms of compassion fatigue was related to the work-based conditions, which was in line with studies that explored the well-being of social workers (Hitchcock et al., 2021).

The studies included in this scoping review recruited from many of the settings (i.e. hospital (Yi et al., 2019)) in which social work is practiced, making it representative of the social work profession.

However, our findings suggested that there were still some working units not included in existing research (e.g., school). To our knowledge, there was no existing research that compared findings across settings or examined the different affect compassion fatigue among social workers in various settings. Future research should explore the incidence across settings and how work settings impacted on the development of compassion fatigue.

Finally, there was a lack of evidence that conducted and tested the effectiveness of interventions that could mitigate compassion fatigue among social workers, who had the unique experiences. Receiving the training or education programs was found to prevent and reduce the increasing symptoms of compassion fatigue among various populations. In contrast to the considerable research dedicated to examining the factors that contributed to compassion fatigue, there were relatively few intervention studies conducted to provide effective interventions and assess their efficacy specifically among social workers, particularly in implementation to other professionals (Delaney, 2018; Slatyer et al., 2017). Instead of focusing on the implement and effectiveness of strategies that reduced compassion fatigue among social workers, some studies only explored strategies themselves through self-reported data and correlational analysis. Therefore, future research should explore more details of the strategies or programs that were applicable to mitigate compassion fatigue among social workers using the quasi-experimental design.

## Limitations

While a systematic process was followed, it was essential to acknowledge that this scoping review might miss certain elements of the literature, especially the missing of relevant articles not included in databases searched. This was a result of limiting the search to English and Chinese literature published within the last two decades. Consequently, the following types of literature were excluded: unpublished (grey literature); published in a language other than English and Chinese; and published as early as 2001. This might introduce some bias, including publication and language.

## Conclusion

In this scoping review, we comprehensively examined the literature on the experiences of CF among social workers and identified five themes, including the prevalence of CF, factors related to CF, attributes and characteristics of CF, consequences of CF, and strategies or interventions to reduce CF among social workers. Although our review provided valuable insights into the CF among social workers, several research gaps remained to be addressed. First of all, the concept analysis and related attributes or theoretical model of CF should to be discussed. And then a more suitable measurement tool for social workers’ experience of CF needed to be developed. Finally, there was a lack of evidence that conducted and tested the effectiveness of interventions that could mitigate compassion fatigue among social workers.
