# Supplementary material for: Understanding compassion fatigue among social workers: a scoping review
Source: Front Psychol. 2025 Jan 27;16:1500305. doi: 10.3389/fpsyg.2025.1500305 (PMC11809495; doi:10.3389/fpsyg.2025.1500305)
Supplement: Supplementary file 2 [file Data_Sheet_2.pdf]

Search Strategy

(1) Database: PubMed

search terms :("social work" OR "social workers") AND ("compassion fatigue" OR "compassion satisfaction" OR "secondary traumatic stress" OR "burnout")

Publication Date: since 2001

(2) Database: ERIC

search terms :("social work" OR "social workers") AND ("compassion fatigue" OR "compassion satisfaction" OR "secondary traumatic stress" OR "burnout")

Publication Date: since 2001

(3) Database: Web of Science

search terms :("social work" OR "social workers") AND ("compassion fatigue" OR "compassion satisfaction" OR "secondary traumatic stress" OR "burnout")

Publication Date: since 2001

(4) Database: EBSCO (Psychology and Behavioral Sciences Collection, APA PsycArticles, APA PsyInfo, and Open Dissertations)

search terms :("social work" OR "social workers") AND ("compassion fatigue" OR "compassion satisfaction" OR "secondary traumatic stress" OR "burnout")

Publication Date: since 2001

（5）CNKI

search terms :("社会工作" OR "社会工作者") AND ("共情疲劳" OR "共情满意" OR "继发性创伤" OR "倦怠")

Publication Date: since 2001

（6）Airiti

search terms :(“社會工作” OR “社會工作者”) AND (“共情疲勞” OR “共情滿意” OR “繼發性創傷” OR “崩熬”)

Publication Date: since 2001
